# Plant Polyisoprenoids and Control of Cholesterol Level

**DOI:** 10.1007/s00005-013-0253-y

**Published:** 2013-08-31

**Authors:** Alexander V. Pronin, Leonid L. Danilov, Alexander N. Narovlyansky, Alexander V. Sanin

**Affiliations:** 1 N. F. Gamaleya Research Institute of Epidemiology and Microbiology, Moscow, Russia; 2 N. D. Zelinsky Institute of Organic Chemistry, Russian Academy of Sciences, Moscow, Russia

**Keywords:** Polyisoprenoids, β-Sitosterol, Sitopren, Cholesterol, Atherosclerosis

## Abstract

The ability of plant polyisoprenoids (polyprenols and polyprenyl phosphates) to diminish the levels of serum cholesterol affecting its biosynthetic pathway are highlighted here. Possible mechanism of such process is discussed. It is also noted that polyisoprenoids can prevent toxic injuries of the liver and restore disturbed hepatic functions. The possibility of polyprenyl phosphates to reveal at the same time anti-inflammatory action suppressing lipoxygenase activity and lowering the levels of proinflammatory cytokines will be illustrated. Attention will be focused on the potential usefulness of plant polyisoprenoids in the course of prevention and treatment of hypercholesterolemia. High efficiency for combined use of polyprenyl phosphate and β-sitosterol, which leads to substantial enhancement of the ability to overcome hypercholesterolemia versus the individual constituents will be demonstrated.

## Introduction

**Fig. 1:**
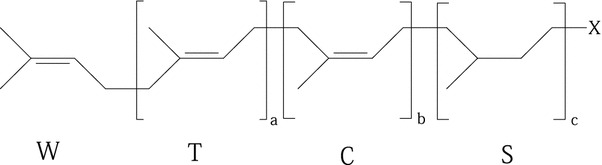
Common formula for polyisoprenoid alcohols and their phosphates. W, ω-isoprene unit; T, *trans*-isoprene unit; C, *cis*-isoprene unit; S, 2,3-dihydroisoprene unit; a, b, and c means a number of T, C, and S-isoprene units, respectively; S = 0 (for polyprenols) or 1 (for 2,3-dihydropolyprenols); X, OH for polyprenols and dihydropolyprenols (dolichols); and X, OPO_3_^2−^ for polyprenyl phosphates and 2,3-dihydropolyprenyl (dolichyl) phosphates

Isoprenoids represents a large and diverse class of naturally occurring organic substances derived from five-carbon isoprene units assembled and modified in diverse ways. Polyisoprenoid alcohols (PI-OH) include polyprenols (PreOH) and dolichols (2,3-dihydropolyprenols, DolOH). PI-OH consists of at least four isoprene units linked head-to-tail (Fig. 1). Molecule of PreOH assembles from *ω*-isoprene unit, two or three *trans*-isoprene units, specified number of *cis*-isoprene units, and *α*-isoprene unit, which has *cis*-configuration and hydroxygroup at C-1 carbon atom; DolOH contain saturated α-unit (IUPAC-IUB Joint Commission on Biochemical Nomenclature 1987). The hydrogenation is catalyzed by steroid 5α-reductase SRD5A3 (Cantagrel et al. 2010). Long-chain linear isoprenoid alcohols are ubiquitous and essential components of cellular membranes of all living organisms (Hartley and Imperiali 2012; Jones et al. 2009; Surmacz and Swiezewska 2011; Swiezewska and Danikiewicz 2005). Polyprenols are isolated mostly from bacteria and green parts of plants, DolOH are typical for fungi and animals. Free PI-OH and their fatty acid esters serve as structural components of cellular membranes modulating their physico-chemical properties such as fluidity and permeability and inducing the formation of non-bilayer zones (hexagonal H_II_ phase) (Chojnacki and Dallner 1988; Hartley and Imperiali 2012; Wang et al. 2008). They are also believed to be the reserve pool of polyisoprenoids for biosynthesis of polyprenyl phosphates in case of need. Phosphates of these PI-OH besides analogous membranotropic behavior serve as the specific membrane-bound intermediary carriers for glycosyl and oligosaccharyl residues in glycan biosynthetic pathways responsible for the production of cellular components such as N-linked glycoproteins or bacterial peptidoglycan (Hartley and Imperiali 2012; Jones et al. 2009; Larkin and Imperiali 2011; Schenk et al. 2001). These functions of PI are of extraordinary significance because genetic disturbances of biosynthetic pathways in which PI and their derivatives participate lead to grave (sometimes fatal) consequences known as congenital disorders (Cantagrel and Lefeber 2011; Cantagrel et al. 2010; Goreta et al. 2012; Grundahl et al. 2012; Kranz et al. 2007). Similar abnormalities are observed at fetal alcohol syndrome particularly as a result of suppression of biosynthesis of dolichol (dolichyl phosphate) from mevalonate by ethyl alcohol (Binghorst et al. 2012).

In animals, PI are synthesized via mevalonate pathway (for recent reviews see, e.g., Buhaescu and Izzedine 2007; Rauthan and Pilon 2011). The mevalonate pathway converts acetyl-CoA to several metabolically important molecules including farnesyl diphosphate (FarPP) and geranylgeranyl diphosphate (GerGerPP) as well as physiologically important end-products. FarPP and GerGerPP are parent compounds for prenylation of proteins particularly G-proteins and earlier viral proteins (Clase et al. 2003; Einav and Glenn 2003; Kapadia and Chisari 2005), and also for biosynthesis of isoprenoid chain of coenzyme Q_10_ (an antioxidant also important in the electron transport chain in mitochondria), PI-OH and their phosphates (important for protein glycosylation). FarPP is a substrate for enzymatic synthesis of cholesterol (important for cell membrane structure and precursor for bile acid and steroid hormones). Thus, cholesterol and polyisoprenoids are biogenetically related compounds (Skorupinska-Tudek et al. 2008). Many enzymes catalyze the multistep pathway, and several therapeutic inhibitors exist that target some of these enzymes, including statins typically prescribed to lower blood cholesterol level. But these therapeutics have several undesired effects. Statins inhibit 3-hydroxy-3-methylglutaryl-coenzyme A (HMG-CoA) reductase, the rate-limiting enzyme in cholesterol biosynthesis, which converts HMG-CoA to mevalonate. Statins lower plasma low density lipoprotein (LDL) cholesterol by causing intracellular cholesterol depletion and upregulating the expression of LDL receptors. Apart from cholesterol, mevalonate is also the substrate for the synthesis of nonsteroid isoprenoids including FarPP, GerGerPP (both attached to small GTP-binding proteins by protein prenyltransferases), coenzyme Q_10_, dolichol, isopentenyladenosine, and so on. Depletion of these isoprenoids results in so-called “pleiotropic” effects of statins, which are independent of cholesterol lowering. Although statins are generally well-tolerated, adverse effects may occur in some patients. These effects result from impaired protein prenylation, deficiency of coenzyme Q_10_ involved in mitochondrial electron transport and antioxidant protection, abnormal protein glycosylation due to dolichol shortage (Beltowski et al. 2009; Rauthan and Pilon 2011).

**Fig. 2:**
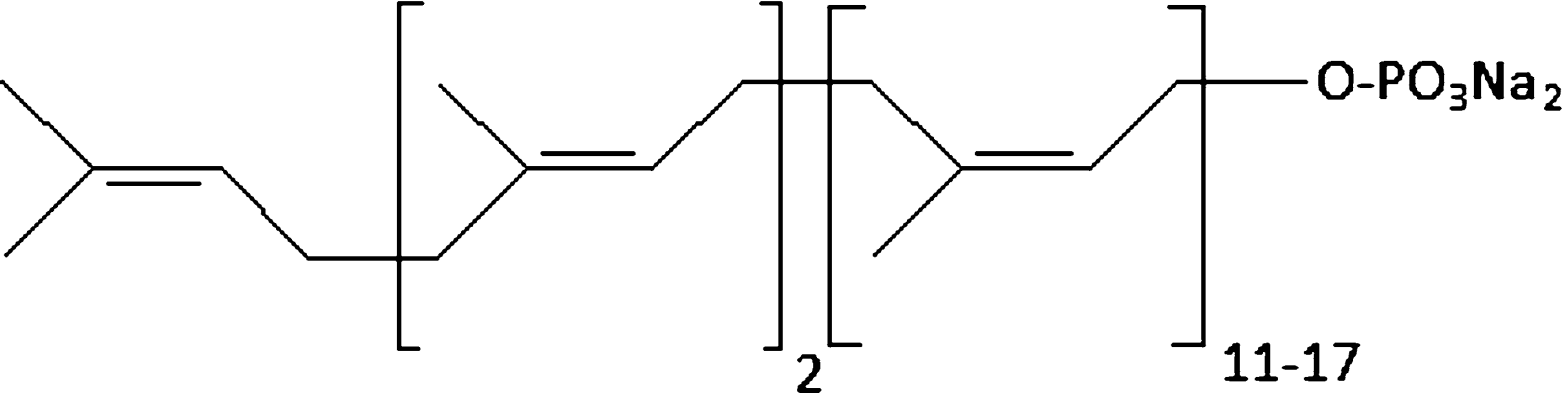
Structural formula of sodium polyprenyl phosphate obtained by phosphorylation of polyprenols from fir (*Abies sibirica*) or pine (*Pinus sylvestris*) needles

**Fig. 3:**
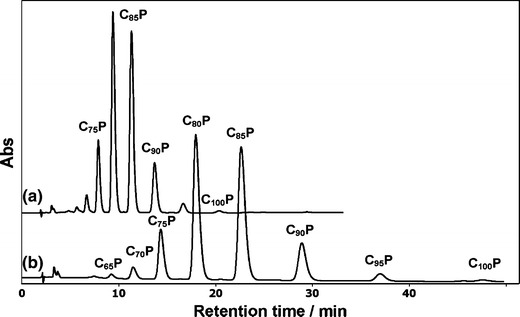
Separation of polyprenyl phosphate oligomerhomologues by reversed-phase ion-pair high-performance liquid chromatography. Typical chromatograms of phosphorylated polyprenols (C_i_P) from fir needles using a reversed-phase Kromasil C18-column 250 × 4.0 mm inner diameter, 5 μm (Eka Chemicals, Sweden) and the mobile phase with the addition 0.020 M tetrabutylammonium dihydrogen phosphate. Conditions: (*a*) ethanol/hexane (87:13 v/v); (*b*) ethanol/hexane (90:10 v/v); flow rate: 0.85 ml/min (from Kozlov and Danilov 2012)

This review is devoted mainly to one of the member of polyisoprenoids-sodium polyprenyl phosphate, which is produced via phosphorylation of PreOH from fir (*Abies sibirica*) or pine (*Pinus sylvestris*) needles. The structure of this substance can be represented by formula (see Fig. 2). Polyprenyl phosphates possess a wide spectrum of physiological effects and seem to be perspective pharmaceutical preparations of a new generation (Danilov et al. 1999, 2003). The first commercial pharmaceutical preparation PHOSPRENYL was developed in Russian Federation and since 1994 has been widely used in the veterinary practice preferably as efficient antiviral drug (Danilov et al. 1996). At the present time, clinical trials of FORTEPREN (the analog of PHOSPRENYL for human) are in progress. The main active ingredient of these preparations is sodium polyprenyl phosphate, which represents the family of oligomerhomologues containing mainly 14–20 isoprene units in the hydrocarbyl radical (Kozlov and Danilov 2012) (Fig. 3).

**Fig. 4:**
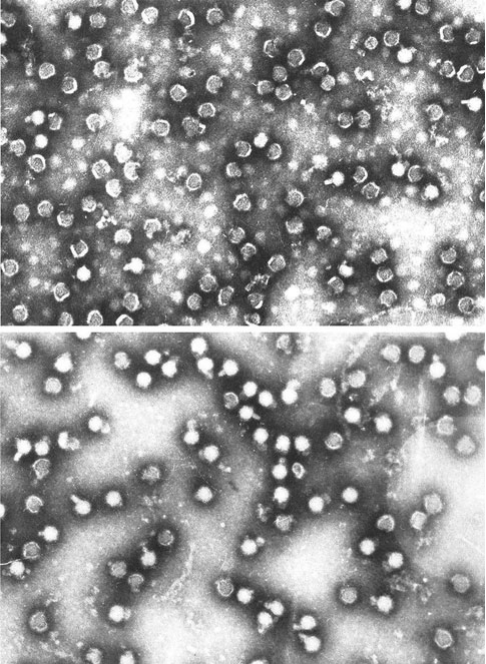
Formation of defected viral particles under influence of sodium polyprenyl phosphate. *Top* *photograph* tick-borne encephalitis virions after 2-h incubation with 200 μg/ml of disodium polyprenyl phosphate (50 μl/ml of 0.4 % w/v solution of the phosphate in complex aqueous solvent containing 3 % v/v of glycerol, 3 % v/v of ethanol and 0.2 % v/v of Tween-80 in water for injection). *Bottom* *photograph* tick-borne encephalitis virions after 2-h incubation with placebo (equal volume of the solvent without the polyprenyl phosphate)

The reason to use polyprenyl phosphates first and foremost as antiviral drug was based upon the data about their ability to exert antiviral action at wide range of serious viral infections such as influenza, herpes simplex, yellow fever, hepatitis, rabies, canine distemper, tick-borne encephalitis, and so on (Danilov et al. 1999, 2003; Ozherelkov et al. 2000; Vasil’ev et al. 2008). The preparations are shown to provoke the production of defected virions, for example, from tick-borne encephalitis virus (strain Soph’in), which was titrated on pig kidney epithelial cell line (SPEV). Electron microscopy morphological study (Fig. 4; negative staining with phosphowolframic acid) revealed that after incubation with polyprenyl phosphate, the majority of virions (65–75 %) had distended or defective envelope. Small viral particles having 20 nm diameter (30–40 nm diameter is normal) were sometimes (5–7 %) found (probably virions lacking envelope). Such virions were not found in control probes. Besides that considerable decrease (relative to control) in number of viral particles was detected in experimental probes. It seems that polyprenyl phosphates disturb the process of viral particle assembly and withdrawal them from the infected cells. But PI possess simultaneously a lot of other physiological properties, which are valuable from medicinal point of view.

## Antihypercholesterolemic and Antihyperlipidemic Action of Polyisoprenoids

It was shown previously (Yamatsu et al. 1986) that in streptozotocin-treated rats, the levels of total cholesterol (TC) and triglycerides (TG) in blood are reliably raised. Injection of dolichol or dolichyl phosphate (15 mg/kg/day) to experimental animals led to substantial lowering of TC and TG levels (TC by 50 % and TG by 75 %).

Recently, it was demonstrated that PreOH are active ingredients of some officinal plants, which are able to ameliorate lipid metabolism, for example, ivy gourd (*Coccinia grandis*) (Tamilselvan et al. 2011). Ivy gourd is a tropical plant used in herbal medicine. Available in supplement form, extracts of the ivy gourd’s roots, fruit, and leaves are known to offer a range of health benefits. Administration of PreOH isolated from *C. grandis* decreased serum TC by 25 % and TG by 42 % in high-fat diet-fed dyslipidemic hamsters at a dose of 50 mg/kg/day. The results were comparable to the action of standard drug fenofibrate at a dose of 108 mg/kg (Singh et al. 2007). Based on these investigations, it can be concluded that PreOH possess marked antidyslipidemic activity.

So, it was revealed that PI display both antiviral properties and the ability to lower the level of cholesterol and triglycerides in blood. Both these effects are seemingly “the links of the same chain”. The confirmation of this supposition is particularly the data obtained by Kuritz (2009). It was demonstrated that addition of polyprenyl phosphates stimulated production of tumor necrosis factor (TNF) in THP-1 cell culture after 3.5 h of such treatment, and antibodies against Toll-like receptor (TLR)2 or TLR4 or their mixture suppressed this stimulation, which means that polyprenyl phosphates were able to interact with TLRs. The increase in intracellular concentration of calcium ions was registered just a few seconds after the addition of polyprenyl phosphate.

**Fig. 5:**
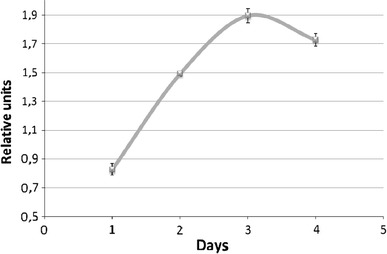
Expression of NFκB by mice splenocytes stimulated with sodium polyprenyl phosphate (RT-PCR). BALB/c mice (≥10 animals in each group) were used. The experimental mice were intramuscularly injected with 20 μg of disodium polyprenyl phosphate (5 μl of 0.4 % w/v solution of the phosphate in complex aqueous solvent containing 3 % v/v of glycerol, 3 % v/v of ethanol and 0.2 % v/v of Tween-80 in water for injection diluted to 0.2 ml with the water) three times every 2 h, the control group animals—with placebo (equal volume of the solvent without the active ingredient). Shown are mean values ± SD; *p* < 0.05

Calcium-mediated signaling is an essential condition for cell stimulation. The next stage of the stimulation is an activation of transcriptional factor NFκB. Figure 5 demonstrates that in 1–3 days after PreP injection in mice expression of NFκB in splenocytes is intensified. One of the final products of these activation are interferons (IFNs).

It was demonstrated that PreP induced the production of type I and II IFN both in vitro and in vivo. Adding polyprenyl phosphate in a dose of 200 μg/ml (in the form of 0.4 % w/v solution of the phosphate in complex aqueous solvent containing 3 % v/v of glycerol, 3 % v/v of ethanol, and 2 % v/v of Tween-80 in water for injection) to the culture of intact murine spleen cells or pig kidney epithelium cells resulted in increasing of IFN titer in the culture liquid to 2.2–3.5 times in comparison with the control cell culture to which the complex solvent without polyprenyl phosphate was added. Infecting the cells with thick-borne encephalitis viruses without adding polyprenyl phosphate gave rise to similar stimulation. Much higher IFN titer increasing (about fourfold) were detected in the cultures treated both polyprenyl phosphate and tick-borne encephalitis viruses. Similar phenomenon was revealed upon analogous adding polyprenyl phosphate to the culture of intact Taurus-1 cells or to the cells infected with infectious bovine rhinotracheitis viruses. So, pronounced interferonogenic activity of polyprenyl phosphate per se and its potentiation of interferonogenic activity of some viruses in vitro were demonstrated (Ozherelkov et al. 2007). Intramuscular injection of 20 μg of disodium polyprenyl phosphate (5 μl of 0.4 % solution of the phosphate in the same complex aqueous solvent diluted to 0.2 ml with water for injection) to BALB/c mice (intact or infected intraperitoneally with tick-borne encephalitis viruses) similarly increased the level of IFN in the blood serum of the animals (Pronin et al. 2012).

As mentioned above, the signal generated after interaction of PreP with TLR2/4 leads to activation of NFκB providing synthesis of type I IFNs. The latter after interaction with receptors IFN-γ receptor 1 induces consecutive activation of Janus kinase 1, tyrosine kinase 2, and STAT1/2, then activation of IFN-stimulated genes and suppression of SREBP2 transcriptional factor (Blanc et al. 2011). This factor (sterol regulatory element-binding protein) is the main regulator of mevalonate pathway producing early precursors of isoprenoids, namely GerPP, FarPP, and GerGerPP. These precursors are also indispensable for prenylation of viral proteins because suppression of their biosynthesis leads to abnormal assembling of viral capsid and forming defective avirulent virions (Clase et al. 2003; Einav and Glenn 2003; Kapadia and Chisari 2005). FarPP is converted into squalene from which cholesterol is produced. FarPP is also shown to be the precursor for de novo-generated polyisoprenoids, which supplement pool of DolOH and their phosphates. SREBP2 provides transcription of practically all key enzymes participating in biosynthesis of cholesterol, such as HMG-CoA synthase, HMG-CoA reductase, FarPP synthase, squalene synthase, and also LDL receptor (Ishimoto et al. 2010; Marquart et al. 2010; Sato 2010). Therefore, suppression of this transcriptional factor activity must considerably inhibits biosynthesis of cholesterol as well as endogenous polyisoprenoid production. But in the last case, possible depletion of the pool could be compensated by means of exogenous polyprenyl phosphate administration.

So, polyprenyl phosphates and statins possess diverse mode of action and do not have common features although formally the therapeutic effect (inhibiting cholesterol biosynthesis) of both of them is the same.

## Anti-inflammatory Activity of Polyprenyl Phosphates

One of the valuable physiological properties of polyprenyl phosphates bearing a relation to atherosclerosis is their anti-inflammatory action. Addition of polyprenyl phosphate to cell culture caused dose-depended decrease of 5- and 15-lipoxygenase activity (Sanin et al. 2011). 5-Lipoxygenase catalyses particularly production of leukotriene B_4_ from arachidonic acid. The leukotriene via interaction with their receptor activate presqualene diphosphate phosphatase, which catalyses conversion of presqualene diphosphate into presqualene monophosphate. Presqualene diphosphate is a potent and direct inhibitor of phospholipase D and phosphatidylinositol-3 kinase whereas presqualene monophosphate is considerably weaker one. Phospholipase D catalyses conversion of membrane phosphatidylcholine into phosphatidic acid and thus regulates such cell functions as morphologic change, degranulation, and O_2_^−^ production. Phosphatidylinositol-3 kinase immediately activates NADPH oxidase—the main inducer of oxidative burst and associated manifestations of inflammatory process (Bonnans and Levy 2007). So, blocking lipoxygenase with polyprenyl phosphate inhibits synthesis of leukotriene B_4_ and following activation of presqualene diphosphate phosphatase. As a result, the active centers of phospholipase D and phosphatidylinositol-3 kinase can remain blocked with presqualene diphosphate, the catalytic activities of the enzymes can remain weak and thereby inflammation can be diminished.

## Hepatoprotective Properties of Polyisoprenoids

One more substantial mode of polyisoprenoids action which has to do with control of cholesterol level is their ability to accelerate regeneration of liver tissue as well as to prevent, normalize and restore hepatic functions. It has been demonstrated (Yamatsu et al. 1986) that regeneration rate of liver in rats with partial hepatectomia was significantly increased by 20 % after injection 30 mg/kg/day of dolichol or by 10 % after injection 15 mg/kg/day of dolichyl phosphate.

Along with dolichol and dolichyl phosphate, plant PreOH and polyprenyl phosphates manifest strongly pronounced complex influence on liver functions. These polyunsaturated substances are biogenetically congeneric to liposoluble vitamins A and E, ubiquinones, and coenzyme Q_10_. They are postulated to possess an antioxidant, antistress, and membranoprotective activities (Bergamini et al. 2004).

The hepatoprotective effects of PreOH from *Ginkgo biloba* L. leaves (GBP) were evaluated against carbon tetrachloride-induced hepatic damage in Sprague-Dawley rats. The effects of GBP were comparable and not significantly different from those of the standard drug Essentiale. The results indicate that GBP could be explored as a potentially promising additive for liver diseases (Yang et al. 2011).

It was demonstrated that taxus polyprenols (TP isolated from the needles of *Taxus chinensis* var*. mairei*) successfully attenuated liver injury induced by CCl_4_ in rats. It was shown by histopathological sections of livers and improved liver function as indicated by lowering alanine transaminase (ALT), aspartate transaminase (AST), and alkaline phosphatase levels, increased albumin levels in serum of the animals and substantial improving other hepatic characteristics. TP also remarkably decreases in malondialdehyde content. These results suggest that the protective effects of TP in chronic CCl_4_-induced liver fibrosis might be related with the reduction of oxidative damage, the inhibition of hepatic stellate cells activation, the down-regulation of pro-fibrogenic stimuli, and the protection of hepatocytes. The activity of TP (similar to GBP) was comparable with such of Essentiale as standard drug (Yu et al. 2012).

It should be mentioned that therapeutical effects were observed after administration of 48–300 mg/kg/day of the PreOH.

The first commercial pharmaceutical preparation Ropren (25 % solution of coniferous PreOH in vegetable oil) was developed in Russia. Recommended doses of Ropren are 72 mg/day for hepatoprotection and 144 mg/day for treatment of alcoholic cirrhosis in men. In the last case, the decrease of TC (by 20 %), ALT, and AST levels was observed (Soultanov et al. 2010). In a model of dichloroethane-induced toxic hepatitis in rat Ropren at a dose 20 mg/kg/day decreased TC (by 10 %), ALT, and AST levels similar to Essentiale; regeneration rate of liver in rats with partial hepatectomia was significantly increased by 10 % after injection of 40 mg/kg/day of Ropren (Roschin and Soultanov 2003).

**Fig. 6:**
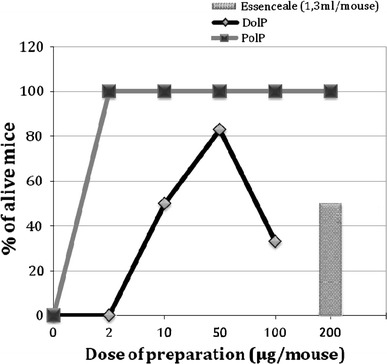
Hepatoprotective effect of sodium polyprenyl or dolichyl phosphate. The preparations were injected intraperitoneally in mice at indicated doses and 1 h later 0.1 ml of CCl_4_ was injected by the same way. The survival of the animals within 4 days was assayed, 10 mice in each group were used. As a reference preparation Essentiale N (Sanofi Aventis, 1.3 ml/mouse) was utilized

We have demonstrated high hepatoprotective action of sodium polyprenyl or dolichyl phosphate in the model of acute toxic hepatitis in mice. Doses 2–200 μg/mouse (0.1–10 mg/kg) of these substances were used for the assays. Single inoculation of the phosphates in mice 1 h before injection of CCl_4_ prevented the fatal outcome much more efficiently than Essentiale (Fig. 6). Dolichyl phosphate at a dose of 50 μg/mouse (2.5 mg/kg) prevented the mortality of animals by 85 % and polyprenyl phosphate completely prevented the mortality while efficiency of Essentiale was only 50 %. It seems therefore that phosphorylated plant polyprenols are substantially more potent hepatoprotectors than PreOH themselves.

## Polyisoprenods as Natural Potential Enhancers and Competitors for Up-to-Date Used Chemotherapeutic Drugs for Prevention and Treatment of Hypercholesterolemia

Summing up the above-mentioned information concerning both biochemical and therapeutical properties of polyisoprenoids, we conclude that these compounds are able to suppress biosynthesis of cholesterol and possess anti-inflammatory and hepatoprotective properties simultaneously. They are non-toxic substances and have practically no side effects (Danilov et al. 1996). Thus, they seem to be well candidates for a new perspective group of preparations for control of blood cholesterol level.

But it is quite evident that such regulation is very delicate and balanced. A suppression of synthesis of cholesterol leads to compensatory increase of its absorption from intestine with NPC1L1 protein (Niemann-Pick C1-like protein 1). In parallel, the activity of ATP-binding cassette transporters (ABCG7/9), which remove synthesized cholesterol from the cells (Tremblay et al. 2011) is blocked. Therefore, in parallel with statins (up-to-date main therapeutic preparations for lowering blood cholesterol level via inhibition of HMG-CoA reductase) ezetimibe (one of cholesterol absorption inhibitors) is frequently used (Sasaki et al. 2011).

The major drawback of using existent anti-cholesterol drugs is related to their side effects. So, development of alternative products which efficiently lower blood cholesterol level and do not inflict injury to organism is very actual task. Plants are the renewable available source of the compounds, which may be applicable for resolving of this problem.

## Phytosterols Application for Lowering Cholesterol Level

As was mentioned above, plant PreOH and polyprenyl phosphates would be potential enhancers (or even competitors) for up-to-date used chemotherapeutic preparations for prevention and treatment of hyperlipidemia. These polyisoprenoids act via suppression of biosynthesis of cholesterol and lowering of cholesterol and triglyceride levels in blood owing to hepatic function normalization, and polyprenyl phosphates seems to be more efficient ones at least as hepatoprotectors.

The second group of natural compounds which is useful for blood cholesterol lowering are plant sterols (phytosterols). They were found to be an attractive alternative to synthetic inhibitors of cholesterol absorption from intestine, and β-sitosterol is most effective one among them (Fernandez and Vega-Lopez 2005). These compounds are now used as food additives and biologically active nutritional supplements. Phytosterols are sterols that are synthesized only in plants and that are structurally similar to cholesterol but with the inclusion of an extra hydrophobic fragment (ethyl radical) at the C24 position. Phytosterols and their esters reduce cholesterol level in the blood in spite of the fact that they are poorly absorbed in intestine. The mechanism by which phytosterols/phytosterol esters interfere with cholesterol absorption is not completely clear, but based on the present understanding, three distinct features have been recognized: (1) physico-chemical effects (e.g., competitive solubilization and co-crystallization); (2) effects at the absorption site (e.g., hydrolysis by lipases and esterases); (3) effects on intracellular trafficking of sterols (Rozner and Garti 2006). Due to poor solubilization of phytosterols in oil and water, they as a rule were used at high doses to achieve a reduction in cholesterol level. The available evidence prove that consumption of about 1.6–2.0 g/day of the compounds preferably dissolved in a fat-rich food matrix is effective in lowering LDL cholesterol levels by 8–10 % (Marangoni and Poli 2010). Phytosterols undoubtedly would be very useful for decrease of cholesterol levels, if their ability to suppress cholesterol absorption could appear at substantially smaller doses achieved by increasing of their bioavailability.

## Combined Use of Polyprenyl Phosphates and β-Sitosterol

Recently, the information about combined use of statins as suppressors of cholesterol biosynthesis and phytosterols as inhibitors of cholesterol absorption from intestine was published, proving increased efficiency for treatment of a hypercholesterolemia (Gupta et al. 2011). It seemed to be only logical to try exchanging statins in this combination by polyisoprenoids in order to eliminate side effects without loss of the efficiency of lowering blood cholesterol level.

That was the reason why original biologically active food supplement (BAFS) containing polyprenyl phosphates and β-sitosterol, and method for their preparation was developed in Russia (Narovlyansky et al. 2011). The BAFS Sitopren contains 1 mg of sodium polyprenyl phosphate and 6.2 mg β-sitosterol as the active ingredients in a unit dose (130 mg tablet).

**Fig. 7:**
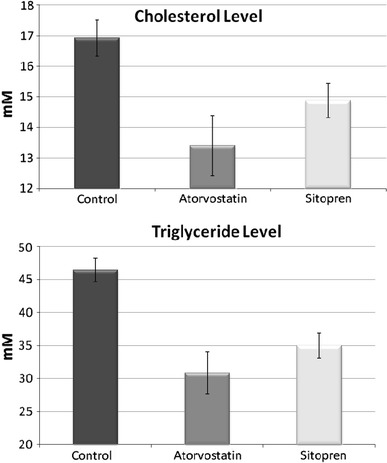
Influence of 50 mg/day (0.4 mg/day of sodium polyprenyl phosphate, 2.4 mg/day of β-sitosterol) of Sitopren or 0.8 mg/day of atorvastatin (each in 0.5 ml of RPMI-1640, Sigma-Aldrich, intragastric administration) on cholesterol and triglyceride levels in serum of hyperlipidemic mice. Control group mice were treated by the same way with 0.5 ml of the diluent without the active ingredient. All mice were injected intraperitoneally with poloxamer-407 (7.5 mg/mouse, every other day, four times) to cause pronounced hyperlipidemia; both control and experimental group contained ≥10 animals. Shown are mean values ± SD; *p* < 0.05

In poloxamer 407-induced model of dyslipidemia (Johnston 2004) in mice, intragastric administration of 50 mg/day of Sitopren (0.4 mg/day of sodium polyprenyl phosphate, 2.4 mg/day of β-sitosterol) significantly decreased the level of serum cholesterol by 12 %. In addition, this BAFS lowered the level of triglycerides by 25 %. These effects were comparable with the action of 0.8 mg/day atorvastatin (Fig. 7). The obtained data unambiguously confirm the antiatherogenic activity of Sitopren.

Peroral administration of 260 mg/day of Sitopren (2 mg/day of sodium polyprenyl phosphate, 12.4 mg/day of β-sitosterol) during 12 weeks diminished TC and LDL levels by 9 % and 12 % correspondingly in a group of volunteers (Narovlyansky et al. 2011). Intake of this BAFS at the same dose and the time interval resulted in significant lowering of TC, TG and LDL levels by 11.4 %, 10.9 %, and 0.6 %, correspondingly, in patients with hypercholesterolemia. The levels of proinflammatory cytokines interleukin (IL)-1, IL-6 and TNF diminished simultaneously by 18.8 %, 16.2 %, and 23.8 %. Index of atherogenicity decreased by 9.7 %. Negative side effects were not revealed (Nebieridze and Chernova 2012).

The results of the assays given above demonstrate high efficiency and safety of Sitopren. This BAFS markedly improves the content of lipids in blood, while significantly decreasing the levels of proinflammatory cytokines, which could be explained by manifestation of its anti-inflammatory and immunomodulatory action. It should be noted that the daily doses of β-sitosterol and polyprenyl phosphate as a constituents of Sitopren (Narovlyansky et al. 2011) were proved to be substantially lower than that of cited in literature for phytosterols (Marangoni and Poli 2010) or PreOH (Roschin and Soultanov 2003; Soultanov et al. 2010) per se for appreciable lowering of cholesterol level in men. The reason of the phenomenon is still not understood and needs further investigation.

These findings form a basis for performing a detailed study of Sitopren action upon the main pathogenetic mechanisms of initiation and development of atherosclerosis such as vascular inflammation and hypercholesterolemia.

## Conclusions

1. Plants are accessible renewable source of a great number of physiologically active substances which are important for human health.
2. Plant polyisoprenoids (PreOH and polyprenyl phosphates) are able to diminish the levels of blood cholesterol affecting its biosynthetic pathway. They also prevent toxic injuries of the liver and restore disturbed hepatic functions. At the same time polyprenyl phosphates express anti-inflammatory activity suppressing lipoxygenase activity and lowering the levels of proinflammatory cytokines.
3. Plant sterols (phytosterols) are natural inhibitors of cholesterol absorption from intestine.
4. Plant polyisoprenoids and phytosterols are non-toxic substances and have practically no negative side effects.
5. These compounds were found to be useful in the course of prevention and treatment of hypercholesterolemia taken separately, or in combination with chemotherapeutic preparations.
6. Combined use of polyprenyl phosphate and β-sitosterol leads to substantial enhancement of the ability to facilitate the initiation and the progress of hypercholesterolemia in comparison with such for phytosterols or PreOH per se. This phenomenon is very valuable for the development of the new type of anti-hyperlipidemic BAFS and preparations consisting exclusively of natural substances like Sitopren.

## Abbreviations

ABCG7/9: ATP-binding cassette (ABC) transporters
ALT: Alanine transaminase
ATP: Adenosine 5′-triphosphate
BAFS: Biologically active food supplement
DolOH: Dolichols
FarPP: Farnesyl diphosphate
GBP: Polyprenols isolated from *Ginkgo biloba* L. leaves
GerGerPP: Geranylgeranyl diphosphate
GerPP: Geranyl diphosphate
GTP: Guanosine-5′-triphosphate
HMG-CoA: 3-Hydroxy-3-methylglutaryl-coenzyme A
IFN: Interferon
IL: Interleukin
LDL: Low density lipoprotein
NADPH: Oxidase, nicotinamide adenine dinucleotide phosphate-oxidase
NFκB: Nuclear factor kappa B
PI: Polyisoprenoids
PI-OH: Polyisoprenoid alcohols
PreOH: Polyprenols
SREBP2: Sterol regulatory element-binding protein 2
STAT1/2: Signal transducer and activator of transcription 1/2
TC: Total cholesterol
TG: Triglycerides
THP-1: Human acute monocytic leukemia cells
TLR: Toll-like receptor
TNF: Tumor necrosis factor
TP: Polyprenols isolated from *Taxus chinensis* var*. mairei* needles
